# Ultrasound sensing with optical microcavities

**DOI:** 10.1038/s41377-024-01480-8

**Published:** 2024-07-09

**Authors:** Xuening Cao, Hao Yang, Zu-Lei Wu, Bei-Bei Li

**Affiliations:** 1 Beijing National Laboratory for Condensed Matter Physics, Institute of Physics, Chinese Academy of Sciences, Beijing, 100190 China; 2 University of Chinese Academy of Sciences, Beijing, 100049 China; 3 School of Optical and Electronic Information, Huazhong University of Science and Technology, Wuhan, 430074 China; 4 Songshan Lake Materials Laboratory, Dongguan, 523808 Guangdong China

**Keywords:** Optical sensors, Microresonators

## Abstract

Ultrasound sensors play an important role in biomedical imaging, industrial nondestructive inspection, etc. Traditional ultrasound sensors that use piezoelectric transducers face limitations in sensitivity and spatial resolution when miniaturized, with typical sizes at the millimeter to centimeter scale. To overcome these challenges, optical ultrasound sensors have emerged as a promising alternative, offering both high sensitivity and spatial resolution. In particular, ultrasound sensors utilizing high-quality factor (*Q*) optical microcavities have achieved unprecedented performance in terms of sensitivity and bandwidth, while also enabling mass production on silicon chips. In this review, we focus on recent advances in ultrasound sensing applications using three types of optical microcavities: Fabry-Perot cavities, π-phase-shifted Bragg gratings, and whispering gallery mode microcavities. We provide an overview of the ultrasound sensing mechanisms employed by these microcavities and discuss the key parameters for optimizing ultrasound sensors. Furthermore, we survey recent advances in ultrasound sensing using these microcavity-based approaches, highlighting their applications in diverse detection scenarios, such as photoacoustic imaging, ranging, and particle detection. The goal of this review is to provide a comprehensive understanding of the latest advances in ultrasound sensing with optical microcavities and their potential for future development in high-performance ultrasound imaging and sensing technologies.

## Introduction

**Fig. 1:**
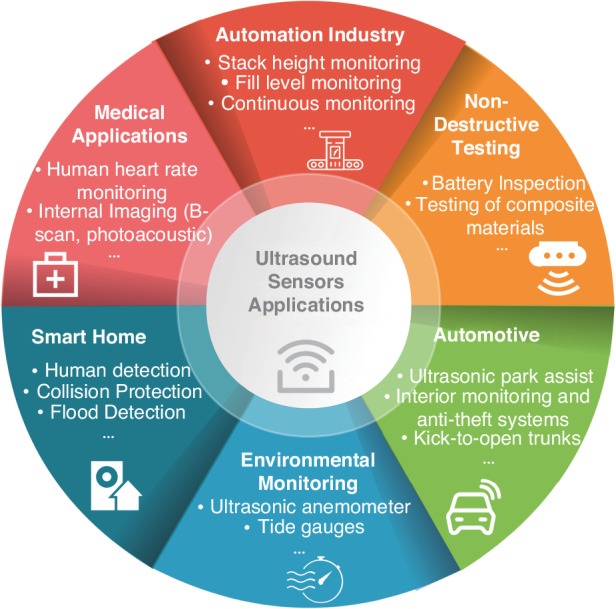
Examples of ultrasound sensor applications

Ultrasound sensing has found widespread applications in various fields, including biomedical imaging^1,2^, industrial non-destructive inspection, and transportation systems. In biomedical imaging, ultrasound stands out for its numerous advantages, including its affordability, ability to provide real-time imaging, and nonionizing radiation. As a result, it has become a commonly used tool for early disease diagnosis^3,4^. Similarly, industries rely on ultrasound technology for applications like flow and level measurement, process control, and non-destructive testing of materials^5^. Furthermore, ultrasound-based systems play a critical role in transportation, facilitating tasks such as reversing radar, object recognition and detection, and automatic obstacle avoidance^6^. All these diverse functions can only be achieved with suitable ultrasound sensors. Figure 1 shows the examples of ultrasound sensor applications.

Piezoelectric transducers have been widely used in industrial and clinical^7^ applications for ultrasound sensing, and have become the predominant ultrasound sensors over the past few decades. These transducers convert ultrasound signals into electric signals by utilizing the piezoelectric effect and measure the electric potential difference resulting from the deformation of the piezoelectric material. However, these transducers have limitations in terms of sensitivity, bandwidth, and miniaturization. Achieving higher frequencies is challenging, and as their size decrease, the sensitivity drops rapidly, resulting in sensor sizes typically in the millimeter to centimeter range. To overcome these limitations, recent advancements in micromachining technology have introduced micro-electro-mechanical systems (MEMS) ultrasound sensors, such as capacitive micromachined ultrasound transducers (CMUTs) and piezoelectric micromachined ultrasound transducers (PMUTs), which offer increased response bandwidth and sensitivity, as well as the potential for integration and miniaturization^8^. The CMUT structure typically comprises a parallel-plate capacitor, with one plate fixed and the other supported by a flexible membrane^9^. An ultrasonic wave causes the membrane to vibrate and leads to a change in the capacitance. These structures generally exhibit significant electromechanical coupling coefficients. Nonetheless, in practical applications, CMUTs often require a high biasing voltage, resulting in substantial power consumption and limited biocompatibility. On the other hand, PMUT harnesses the piezoelectric effect for ultrasound sensing, offering a low-cost technology and requiring low power^10–12^. However, its performance is notably influenced by the characteristics of the piezoelectric material and the residual stress present in the transducer. Both CMUTs and PMUTs are susceptible to electromagnetic interference due to material properties and sensing mechanism, and their opaque sensor structures present challenges for multimodal imaging.

**Fig. 2:**
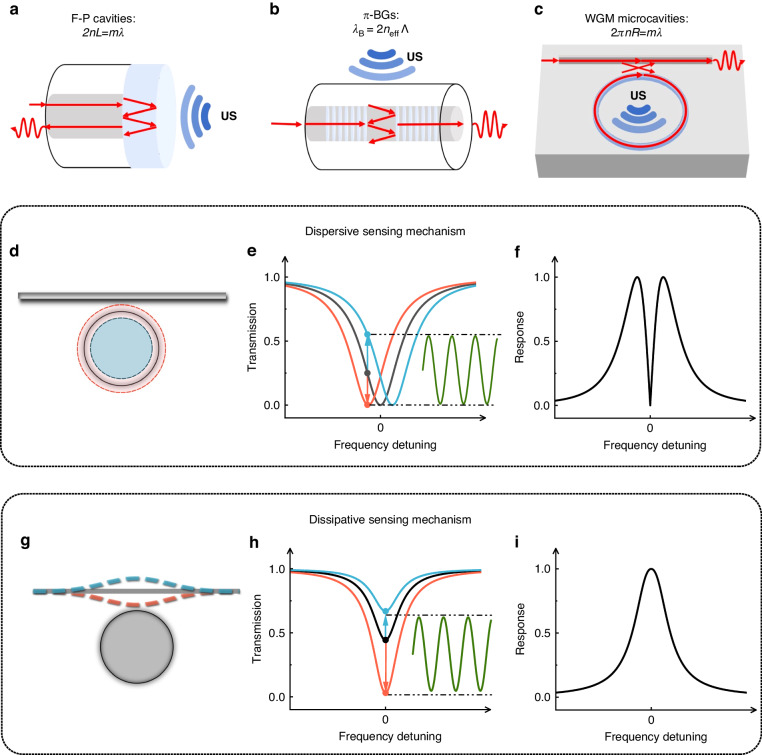
Different types of optical microcavitiy ultrasound sensors and sensing mechanisms. **a**–**c** Schematic illustrations of three types of microcavities for ultrasound sensing: F-P cavity (**a**), π-BG (**b**), and WGM microcavity (**c**), along with their respective resonance condition. **d**–**i** Dispersive (**d**–**f**) and dissipative (**g**–**i**) sensing mechanisms. **d**, **g** Conceptual schematics of the WGM cavity-based dispersive and dissipative sensors. **e**, **h** Cavity transmission spectrum changes in the presence of dispersive and dissipative coupling, respectively. **f**, **i** Responses of the dispersive and dissipative sensing mechanisms, respectively, as functions of the frequency detuning of the input laser from the cavity resonance

In recent years, optical ultrasound sensors have emerged as a promising direction in ultrasound sensing, offering enhanced sensitivity^13–15^ and integration capability. These sensors have undergone continuous miniaturization, transitioning from free-space optical paths to optical fiber paths and now to on-chip integration processes. Optical ultrasound sensors can be classified as resonance-based or non-resonant-based, depending on their measurement approach^16^. Non-resonant-based methods, such as Michelson interferometers^17^, utilize interference to measure ultrasound by monitoring the interferometric phase change resulting from the change of optical path caused by the ultrasound. Early Michelson interferometric ultrasound sensing is available in free-space systems. To enhance the portability and practicality, optical fibers^18–20^ and waveguide structures^21,22^ have been widely employed. Moreover, optical microcavities, such as Fabry-Perot (F-P) cavities, π-phase-shifted Bragg gratings (π-BGs), and whispering gallery mode (WGM) microcavities, have been utilized to further improve ultrasound sensitivity^23^, with the schematics along with their resonance conditions illustrated in Fig. 2a–c. These optical microcavities undergo changes in their refractive index, radius, or waveguide-cavity coupling distance in response to the ultrasound. By monitoring the resulting shift in resonance frequencies or changes in coupling strength, ultrasound can be detected using the dispersive (Fig. 2d–f) or dissipative (Fig. 2g–i) sensing mechanisms, respectively. The high-*Q* optical resonances of microcavities enable ultrahigh measurement precision, offering unprecedented ultrasound sensitivity. Additionally, the mass production capability of microcavities on silicon chips can reduce costs, while their microscale sizes allow for high spatial resolution, particularly in applications like photoacoustic tomography. In the past few decades, various ultrasound sensing applications have demonstrated the potential of optical microcavities.

In this review, we provide an overview of ultrasound sensing using optical microcavities, including the sensing mechanisms and key parameters relevant to ultrasound sensors. Understanding these sensing principles is crucial for comparing the performance of different sensors. We then highlight recent influential research in this field, focusing on the three types of microcavities: F-P cavities, π-BGs, and WGM microcavities. We summarize their key parameters, including bandwidth and sensitivity, and compare their respective advantages and disadvantages. Furthermore, we examine the performance of these microcavity-based sensors in practical applications. Finally, this review presents a comprehensive comparison of ultrasound sensors based on optical microcavities and provides insights into their future development.

## Ultrasound sensing mechanism

The F-P cavity, also known as the F-P interferometer or etalon, is the most commonly used microcavity. It consists of two parallel reflecting surfaces, or thin mirrors, that can confine light in between. The cavity is named after Charles Fabry and Alfred Perot, who created the instrument in 1899. The resonance condition occurs when the optical path in one roundtrip equals an integer number of the light wavelength: 2*nL* = *mλ*, as shown in Fig. 2a. F-P cavities are widely utilized in lasers, telecommunications, optical instruments, spectroscopy, astronomy, etc., due to their high-*Q* factors and well-established fabrication techniques. They have also found significant applications in ultrasound sensing, as ultrasound can alter the cavity length and shift the optical resonance frequency, which can be optically detected. F-P cavities have a simple structure and demonstrate excellent sensitivity when using a thin film on one side of the cavity. F-P cavities located at the fiber end can serve as probe-type ultrasound receivers, and clustering multiple optical fibers can enable array sensing. However, F-P cavities generally have a larger volume compared to other optical cavities. More recently, ultrasound sensors based on optical fibers containing fiber Bragg gratings (FBGs) have been developed, offering advantages of cost-effectiveness and remote-sensing capabilities. Among these FBGs, π-phase-shifted FBGs are particularly intriguing to researchers. These FBGs have a notch in their transmission spectrum that arises from a π-phase discontinuity in the center of the grating. By introducing a π-phase shift into a refractive index modulation of the FBG during its fabrication, a narrow bandpass resonance of a few picometers appears within the middle of the reflection lobe. This narrow linewidth enables highly sensitive ultrasonic detection, addressing the sensitivity limitations of standard FBGs. The resonance condition for π-phase-shifted FBGs is expressed as *λ*_B_ = 2*n*_eff_Λ, where Λ is the grating period of the FBG, as shown in Fig. 2b. In recent years, Bragg gratings on chip-integrated waveguides have also been developed and applied in ultrasound sensing. π-phase-shifted Bragg gratings (π-BGs) feature a small sensing area and can be integrated on-chip or on optical fibers seamlessly. Nevertheless, it is worth noting that their current sensitivity levels are relatively lower. In addition to the F-P cavities and π-BGs, ultrasound sensors based on WGM microcavities have also gained increasing interest, owing to their advantages of high optical *Q* factors and chip-integration capabilities. These WGM microcavities confine light through continuous total internal reflection along the inner surface of a closed circular dielectric structure. The resonance condition for WGM microcavities is satisfied when the optical path equals an integer number of the wavelength: 2*πnR* = *mλ*, where *n* is the effective index, *R* is the radius of the cavity, and *m* is an integer number, as shown in Fig. 2c. WGM microcavities possess high optical *Q* factors and small modal volumes, as well as other advantages such as adaptability to various material systems and geometric shapes. These microcavities can achieve high sensitivities and large bandwidths for ultrasound detection in different systems, making them versatile and suitable for various applications. However, practical applications of WGM microcavities have been hampered by the complexity of their fabrication process and the challenges associated with integration.

When the optical field is resonant with the cavity mode, a Lorentzian-shaped resonance dip appears in the transmission spectrum. The linewidth of the resonance depends on the optical quality (*Q*) factor of the cavity mode and is determined by the optical losses of the cavity. A smaller optical loss results in a higher optical *Q* factor (denoted with *Q*_o_) and a narrower resonance linewidth *δω*, which can be quantified by *δω* = *ω*/*Q*_o_, with *ω* being the resonance angular frequency. The optical *Q*_o_ factor can also be expressed as *Q*_o_ = *ω*/*κ*, with *κ* being the optical decay rate of the cavity mode. A higher *Q*_o_ is desirable for sensing, as it provides a higher phase measurement precision. The depth of the resonance dip is determined by the coupling strength between the waveguide and the cavity. In the presence of an ultrasonic wave, the ultrasound pressure can induce changes in the optical characteristic through two distinct mechanisms. Firstly, it can induce an optical resonance shift by altering the refractive index through the photoelastic effect or changing the cavity radius by exerting a force on the cavity. Alternatively, it can modify the coupling strength by changing the gap between the coupling waveguide and the cavity. Both the optical resonance shift and the change in the coupling strength will induce a variation in the intracavity optical field, which can be converted into an electric signal using a photodetector. In the case of relatively small acoustic signals, the mode changes caused by the acoustic pressure can be regarded as linear changes. Consequently, the frequency of the detected electric signal is the same as the ultrasound frequency, and the signal amplitude is proportional to the ultrasound pressure. The temporal signal of the acoustic wave can be captured using an oscilloscope, and applying the Fourier transform or employing a spectrum analyzer enables the acquisition of the frequency-domain signal. The sensing mechanisms that rely on the optical resonance shift and change in the coupling strength are referred to as dispersive and dissipative sensing mechanisms, respectively.

### Dispersive sensing mechanism

The dispersive sensing mechanism is one of the most commonly used sensing mechanisms for microcavity ultrasound sensing^24^. The principle of this mechanism is illustrated in Fig. 2d–f. When ultrasound is incident on a microcavity, the resonance frequency shifts due to the refractive index change caused by the photoelastic effect and cavity length variation induced by stress (Fig. 2d). This translates into a periodic modulation of the intracavity optical field at the ultrasound frequency. In the measurement, the frequency of the laser is usually locked to the side of the optical resonance to measure the amplitude modulation induced by the ultrasonic wave (Fig. 2e). The optical readout response is proportional to the slope of the transmission, as shown in the response as a function of the optical frequency detuning in Fig. 2f. As a result, having a higher optical *Q* factor is desirable to achieve higher readout sensitivity. The maximum response is obtained when the frequency detuning $\delta \omega =\sqrt{3}\kappa /6$. The dispersive sensing mechanism can also be read out by locking the laser frequency at the center of the optical resonance and measuring the phase modulation. An interferometer is often used to measure the phase modulation^25^. The laser phase noise can be reduced by balancing the two interferometric arms.

### Dissipative sensing mechanism

Unlike the dispersive sensing mechanism that measures mode shift, the dissipative sensing mechanism relies on the change in the optical linewidth to read out the ultrasound, as shown in Fig. 2g–i. Ultrasound changes the total decay rate *κ* by varying the rate of optical coupling into the cavity *κ*_1_ or the intrinsic decay rate of the cavity *κ*_0_ (Fig. 2g). The variation in the decay rate leads to changes in the coupling depth and thus the output light intensity, as well as the linewidth of the optical mode. The optical intensity change modulated by ultrasound can be read out by locking the incident light frequency on the optical resonance (Fig. 2h). The response decreases when the detuning increases and reaches the maximum when the detuning *δω* = 0 (Fig. 2i). The advantage of the dissipative sensing mechanism is that some optical microcavities are not very susceptible to cavity length changes, and measuring the coupling rate changes between the cavity and the coupling waveguide can improve the response to ultrasound. For instance, a recent study by Meng et al.^26^ found that the perimeter of the microsphere does not change significantly under ultrasound. Instead, due to the large optical field gradient between the fiber taper and the microsphere, measuring the intensity change through the dissipative coupling sensing mechanism can effectively enhance the sensitivity.

## Key parameters of ultrasound sensors

There are various parameters to evaluate the performance of ultrasound sensors, such as sensitivity, responsiveness, center frequency, bandwidth, spatial sensing capability, stability, size, etc. Different aspects are emphasized for comparison based on various application requirements. In the following, we will focus on three key parameters of ultrasound sensors: sensitivity, working frequency and bandwidth, and spatial sensing capability. These parameters are more commonly used in ultrasound sensing applications.

### Sensitivity

Sensitivity is a critical parameter for ultrasound sensors as it determines their ability to detect weak ultrasonic waves. It is defined as the smallest detectable ultrasound pressure. In the case of optical ultrasound sensors that use light intensity to read out the signal, sensitivity is typically characterized by the noise equivalent pressure (NEP), which represents the amplitude of ultrasound pressure that can be detected by the sensor at a signal-to-noise ratio (SNR) of 1. By calibrating the system noise to the effective pressure incident at the sensor surface, NEP allows for accurate sensitivity characterization. It is important to consider the bandwidth of the incident sound pressure, as NEP (measured in Pascal) denotes the amplitude of the sound pressure within a specific bandwidth. To evaluate the sensitivity of ultrasound sensors within a unit bandwidth, the noise equivalent pressure density (NEPD) can be utilized^27^. It is measured in Pa Hz^−1/2^, and represents the NEP for a bandwidth of 1 Hz, corresponding to a measurement time of one second. Increasing the measurement time reduces the noise floor and therefore improves the NEP. It should be noted that NEP and NEPD are sometimes used interchangeably without explicit differentiation in some articles.

To enhance the ultrasound sensitivity of optical microcavities, mechanical resonances can also be employed, which can further enhance the response to external stimuli by a factor of *Q*_m_, with *Q*_m_ denoting the mechanical quality factor. The strong optomechanical coupling enables optical readout of the mechanical displacement. In the past few decades, optomechanical systems have been extensively applied for sensing of multiple physical quantities^28–30^, such as displacement^31–33^, force^34,35^, mass^36,37^, acceleration^38^, magnetic field^39,40^, ultrasound^41,42^, etc. In the following, we use a cavity optomechanical system to interpret the sensitivity.

The sensitivity of ultrasound sensors is ultimately determined by the noise level of the system. In cavity optomechanical sensors, the main sources of noise include thermal noise, which is related to the environment temperature, and detection noise from the probe laser. Thermal noise arises from the environmental medium damping and intrinsic structural loss, and its displacement noise power spectral density (PSD) is expressed as^43^

1 $${S}_{xx}^{{{{\rm{thermal}}}}}(\omega )=| \chi (\omega ){| }^{2}{S}_{FF}^{{{{\rm{thermal}}}}}=\frac{2\gamma {k}_{{{{\rm{B}}}}}T}{\left.m\left[{\left({\omega }_{{{{\rm{m}}}}}^{2}-{\omega }^{2}\right)}^{2}+{\omega }^{2}{\gamma }^{2}\right)\right]}$$

**Fig. 3:**
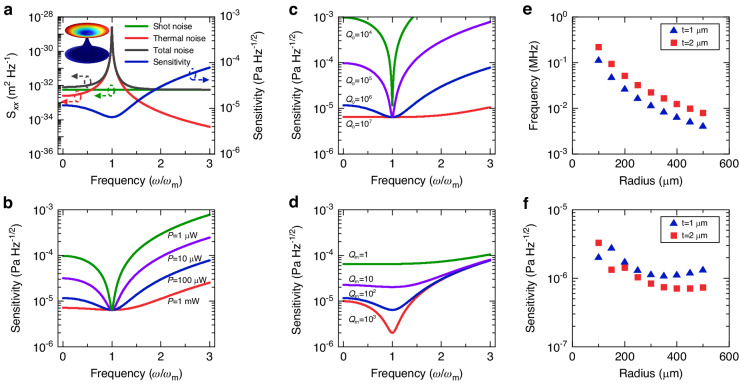
Sensitivity analysis. **a** Displacement PSDs for the thermal noise (red curve), shot noise (green curve), total noise (black curve) on the left axis, and the corresponding sensitivity spectrum (blue curve) on the right axis. The inset shows the simulated displacement distribution of the first-order flapping mode. **b** Sensitivity spectra of the microdisk ultrasound sensor, at incident optical powers of 1 μW (green curve), 10 μW (purple curve), 100 μW (blue curve), and 1 mW (red curve), for *Q*_o_ of 10^6^. **c** Sensitivity spectra for different optical quality factors *Q*_o_, at an incident optical power of 100 μW. **d** Sensitivity spectra at different mechanical quality factors *Q*_m_ = 1 (green curve), *Q*_m_ = 10 (purple curve), *Q*_m_ = 100 (blue curve), and *Q*_m_ = 1000 (red curve). The microdisk ultrasound sensor used here has a radius of 100 μm and a thickness of 2 μm. The frequency of the first-order flapping mode is 1.3 MHz. **e** Simulated mechanical resonance frequencies of the flapping modes of 1 μm-thick and 2 μm-thick microdisks respectively, as a function of the disk radius. **f** Calculated sensitivities of microdisks, with thicknesses of 1 μm and 2 μm, respectively. The blue triangles and red squares represent the results of the first-order flapping modes of the microdisk with thicknesses of 1 μm and 2 μm

Here, *χ*(*ω*) = $\frac{1}{m({\omega }_{{{{\rm{m}}}}}^{2}-{\omega }^{2}-i\gamma \omega )}$ represents the mechanical susceptibility, quantifying the displacement of the mechanical resonator in response to an external force in the frequency domain, for a simple case of a single mechanical resonance with an angular frequency of *ω*_m_. The parameters *m* and *γ* represent the effective mass and damping rate of the mechanical resonator, respectively. Decreasing *γ* (increasing mechanical quality factor *Q*_m_) can enhance the response to near-resonant forces. The detection noise includes classical technical noise (phase noise and intensity noise) and quantum shot noise. The technical noise can be significantly suppressed by using homodyne or heterodyne detection schemes. Consequently, we only consider shot noise here^29^. To better visualize the noise spectrum and sensitivity as a function of the frequency, a microdisk optomechanical sensor is utilized as an example. The microdisk has a radius of 100 μm and a thickness of 2 μm, with a simulated mechanical resonance frequency of 1.3 MHz. The red curve in Fig. 3a shows the thermal noise PSD near the mechanical resonance frequency, with the inset displaying the simulated displacement distribution of the first-order flapping mode. The temperature *T* is 300 K and the *Q*_m_ is 100. It can be observed that there is a thermal noise peak near the mechanical mode due to resonance enhancement, with the response at the mechanical resonance being enhanced by a factor of 100. The displacement PSD of the shot noise is expressed as^44^

2 $${S}_{xx}^{{{{\rm{shot}}}}}(\omega )=\frac{\kappa }{16\eta N{G}^{2}}\left(1+4\frac{{\omega }^{2}}{{\kappa }^{2}}\right)$$

In this equation, $N={Q}_{{{{\rm{o}}}}}P/\hslash {\omega }_{{{{\rm{L}}}}}^{2}$ is the intracavity photon number, where *P* is the incident optical power and *ω*_L_ is optical resonance frequency. *κ* = *ω*_L_/*Q*_o_ is the optical power decay rate, and *η* stands for the optical detection efficiency. $G=\frac{d\omega }{dx}$ represents the optomechanical coupling coefficient, quantifying the optical resonance frequency shift for a mechanical displacement *x*. The shot noise PSD is depicted by the green curve in Fig. 3a, where the optical power is *P* = 100 μW and optical *Q* factor *Q*_o_ = 10^6^. The shot noise remains constant within the frequency range and only increases significantly when the frequency is comparable to *κ*/2*π*. The total noise, which consists of the sum of thermal noise and shot noise, is plotted in the black curve in Fig. 3a, indicating that the total noise is dominated by thermal noise near mechanical resonance frequency and by shot noise when it is far from the mechanical resonance.

The sensitivity (or NEPD) can be obtained from the noise PSD, which is calculated using the following equation:

3 $${{{\rm{NEPD}}}}=\frac{1}{r\zeta A}\sqrt{\frac{{S}_{xx}^{{{{\rm{shot}}}}}}{{\left\vert \chi \right\vert }^{2}}+{S}_{FF}^{{{{\rm{thermal}}}}}}=\frac{1}{r\zeta A}\sqrt{\frac{\kappa }{16\eta N{G}^{2}{| \chi | }^{2}}[1+4{\left(\frac{\omega }{\kappa }\right)}^{2}]+2m\gamma {k}_{{{{\rm{B}}}}}T}$$

Here, *r* represents the ratio of the pressure difference between the upper and lower surfaces of the sensor to the peak pressure at the antinode of the incident ultrasonic wave, *ζ* is the spatial overlap between the incident ultrasound and the mechanical displacement profile of the sensor, *A* is the sensor area. The sensitivity as a function of the frequency is shown in the blue curve in Fig. 3a. It can be seen that the sensitivity reaches a minimum at the mechanical resonance frequency where thermal noise dominates and is degraded in the shot-noise-limited regime. This is due to the fact that the mechanical resonance not only enhances thermal noise but also enhances response. However, shot noise is not amplified by the mechanical resonance. Therefore mechanical resonance helps to increase the SNR. The thermal-noise-limited sensitivity represents the fundamental limit for ultrasound sensors. Consequently, reaching this limit is critical to achieving high sensitivity for ultrasound sensors.

The thermal-noise-dominant regime can be reached by optimizing the parameters to reduce shot noise or increase thermal noise. Equation (3) shows that increasing the probe power *P*, optical quality factor *Q*_o_, or the optomechanical coupling coefficient *G*, can reduce the contribution of shot noise. Figure 3b, c show the sensitivity spectra for various incident powers when the *Q*_o_ is fixed at 10^6^, and for different *Q*_o_ when the incident power is fixed at 100 μW, respectively. Both incident power and *Q*_o_ have no effect on the thermal noise term, so the minimum NEPD (sensitivity at the mechanical resonance frequency) achievable by the system remains constant regardless of variations in these two parameters. Both Fig. 3b and 3c demonstrate that as *P* and *Q*_o_ increase, the shot noise decreases, and the frequency range of the thermal noise dominant regime increases, thus extending the detection bandwidth. It is evident that the sensitivity exhibits a flat spectrum within a frequency range of approximately *ω*_m_, given an appropriate selection of *P* and *Q*_o_. Moreover, as ${S}_{xx}^{{{{\rm{shot}}}}}\propto \frac{1}{{Q}_{{{{\rm{o}}}}}^{2}}$ and ${S}_{xx}^{{{{\rm{shot}}}}}\propto \frac{1}{P}$, increasing the *Q*_o_ leads to a more effective reduction of the shot noise than increasing the incident power.

Another way to achieve the thermal-noise-dominant sensitivity is by improving the mechanical quality factor *Q*_m_. Increasing *Q*_m_ can improve the thermal-noise-dominant sensitivity, as shown in Fig. 3d. However, increasing *Q*_m_ will also lead to a narrower linewidth of the mechanical peak and therefore the thermal-noise-dominated frequency range (i.e., the bandwidth). Due to the high *Q*_o_ = 10^6^, the thermal-noise-dominated regime can still be reached even when the *Q*_m_ = 1. Given this scenario, a microcavity with a lower *Q*_m_ can realize broadband detection, although at the expense of compromised sensitivity. On the other hand, a microcavity with higher *Q*_m_ can achieve better sensitivity but with a limited bandwidth. These findings highlight that both optical resonance and mechanical resonance can enhance sensitivity from different perspectives. The dual resonance in the cavity optomechanical system enables extremely high sensitivity and has found widespread applications in the measurement of various physical quantities^29^.

Equation (3) also suggests that the sensitivity improves with a larger sensor area *A*. However, the effect of the pressure difference needs to be taken into account. Figure 3e displays the simulated resonance frequencies of the first-order flapping mode of the microdisk as a function of the disk radius, considering thicknesses of both 1 μm (blue triangles) and 2 μm (red rectangles), respectively. It is observed that the resonance frequency decreases with increasing radius and decreasing thickness. To evaluate the impact of these parameters on sensitivity, we obtain the spatial overlap and pressure difference through simulation, and calculate the corresponding sensitivities for microdisks with different radii and thicknesses of 1 μm and 2 μm, as shown in Fig. 3f. According to Eq. (3), increasing the radius and decreasing the thickness improves the sensitivity due to increased sensor area or reduced mass. However, the decrease in resonance frequency hinders the sensitivity improvement due to the reduced pressure difference. As a result, considering the combined effect of these two factors, the sensitivity initially improves and then degrades with increasing radius. Moreover, the sensitivities of 2 μm-thick microdisks are generally better than those of 1 μm-thick microdisks at most radii.

### Working frequency and bandwidth

Ultrasound is a type of acoustic wave that operates above 20 kHz and has a wide range of frequencies. The working frequency and bandwidth of ultrasound sensors are crucial factors as they determine the applications for which the ultrasound can be used. In the field of ultrasound imaging, higher frequencies are preferred as they provide better spatial resolution. To achieve micrometer-level spatial resolution, ultrasound sensors need to have a center frequency and bandwidth in the MHz range^9^. However, it is important to seek a balance between high frequencies and the loss of ultrasound waves in the medium. As the frequency increases, so does the absorption and scattering loss in the medium. The absorption loss is directly proportional to the frequency, while the scattering loss is proportional to the frequency squared^6^. In the case of ultrasonic waves in the air, the scattering loss dominates, with an attenuation of approximately 160 dB/m for a 1 MHz ultrasound. Therefore, it is necessary to consider both the penetration depth and image resolution when selecting the frequency of ultrasound waves. In other applications, such as thermoacoustic and photoacoustic reconstruction^45^, the detection bandwidth plays a crucial role in determining the axial resolution (*R*_*A*_), which can be described by the equation *R*_*A*_ = 0.88*v*_*A*_/*BW*, where *v*_*A*_ represents the speed of sound, and *BW* denotes the bandwidth of the detector. Besides, the lateral resolution of photoacoustic imaging depends on the beam waists of the optical or acoustic focal points^46,47^. A wider bandwidth allows for more detailed detection in three dimensions. Additionally, in applications like ultrasonic ranging where the time-of-flight (TOF) method is used to determine the position by reflecting sound waves, a larger bandwidth leads to a narrower pulse width in the time domain, resulting in higher precision. In specific applications like sonar and underwater communications, kHz frequency acoustic sensors are required to minimize acoustic loss and extend the detection and communication ranges.

The bandwidths of traditional piezoelectric transducers typically range in the megahertz level, with center frequencies between 1 MHz and 100 MHz and fractional bandwidths (the ratio between the − 3 dB or − 6 dB bandwidth and the center frequency) of 60–80%. However, capacitive or piezoelectric micromachined ultrasound transducers can achieve a fractional bandwidth over 100%, albeit with compromised center frequency in the few megahertz range^48^. A bandwidth of up to several hundred megahertz can be achieved using optical microcavity ultrasound sensors^49^. For optical resonance-based ultrasound sensors, the intracavity photon lifetime is one of the factors limiting the bandwidth. A lower optical *Q* factor corresponds to a shorter photon lifetime and a broader bandwidth. Consequently, there is a trade-off between sensitivity and bandwidth, regarding the choice of optical *Q* factor. Mechanical resonances can enhance the cavity response, and the bandwidth is related to the range dominated by thermal noise. In the unresolved sideband regime (*κ* > *ω*_m_), it is possible to increase the thermal noise dominant frequency range, thereby improving the bandwidth by enhancing the optical *Q* factor or increasing the incident optical power. In contrast, for microcavity ultrasound sensors without mechanical resonances, such as microrings^49^ and F-P cavity sensors^50^, their response bandwidths depend mainly on the thickness of the microcavity and substrate as well as the acoustic impedance.

### Spatial sensing capability

The spatial sensing capability of ultrasound sensors includes the ability to detect ultrasonic waves from different directions (known as the acceptance angle) and at different distances. The shape and size of the sensor play a significant role in these capabilities. Typically, ultrasound sensors are most sensitive to axial ultrasound, and their sensitivity decreases as the incidence angle. Piezoelectric transducers, commonly used in ultrasound sensors, have a directional nature with acceptance angles usually below ± 20^∘^^51^. Acoustic lenses may be required to increase their acceptance angles. In imaging applications, a wider acceptance angle is desirable to capture more realistic spatial information, making optical ultrasound sensors more advantageous. Various types of optical microcavity ultrasound sensors exist, with some capable of achieving almost full-angle response^52^. Spherical sensors, especially those considered point-like, exhibit a larger acceptance angle. In contrast, microdisk or membrane sensors, have a limited spatial angular response range, especially at higher frequencies^53^. Furthermore, the detection distance of the sensor is also a critical factor. In scenarios where the sensor can be treated as a point, ultrasound sensing at far distances may be weakened due to insufficient sensing area and associated propagation losses. To minimize ultrasound propagation loss, sensors are often placed in proximity to acoustic sources. However, this near-field detection approach comes with its drawbacks. When the detection distance is comparable to the size of the sensor, acoustic waves reaching different locations on the sensor will undergo phase retardation, thereby influencing the response. Compared with microdisks, the ring shape has a clear advantage in near-field ultrasound detection because the geometric simplicity minimizes the phase retardation^53^. While placing the microring cavity ultrasound sensors in the acoustic far field provides a longer working distance and a broader acceptance angle, detection in the acoustic near field offers improved sensitivity and broader bandwidth but at the expense of a reduction in the acceptance angle^54^.

## Optical microcavity ultrasound sensors

In this section, we present the working principles, recent research progress, and applications of the above-mentioned three types of microcavity ultrasound sensors.

### Fabry-Perot cavity ultrasound sensors

**Fig. 4:**
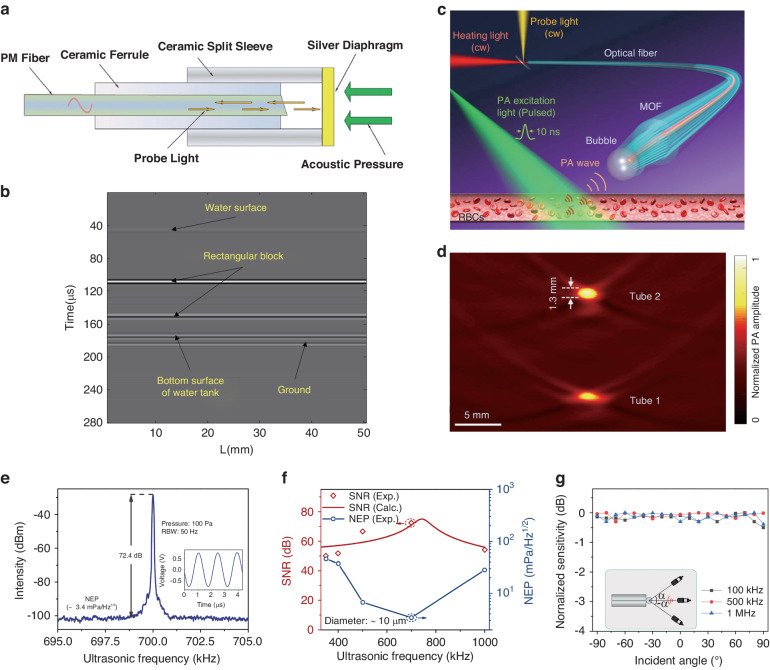
Different types of F-P cavities for ultrasound sensing. **a** Schematic of the sensing head based on a large area silver diaphragm. **b** A rectangular Plexiglas block ultrasound image reconstructed using the TOF approach. **c** Schematic of a surface microbubble photothermally generated at a microstructured optical fiber tip for photoacoustic imaging of red blood cells in a blood vessel. **d** Reconstructed cross-sectional image of the blood-filled tubes. **e** Ultrasound response of a 10 μm diameter microbubble to the sinusoidal ultrasound wave. Inset: ultrasound response in the time domain. **f** SNR and NEP versus the ultrasound frequency. **g** Directivity of the microbubble for ultrasound waves at different frequencies. Reprint **a** with permission from ref. ^62^ ©Optica Publishing Group; (**b**) from ref. ^64^; (**c**–**g**) from ref. ^65^

The F-P cavities are the most fundamental type of optical resonators and are widely used in numerous sensors^35,55–59^. These cavities employ two highly reflective mirrors to confine light between them, which can be created either utilizing free space light propagation, optical fibers, or chip-integrated structures. A majority of ultrasound sensors based on F-P cavities are created at the end of an optical fiber, with one mirror replaced with a highly reflective film to improve both the optical *Q* factor and the response to ultrasound^60^. Ultrasound incident on the film causes a change in cavity length, thus modulating the intensity of the reflected light. In 2013, an F-P cavity using a multilayer graphene film as a reflector was used for ultrasound sensing^61^. Using a thin film of only 100 nm thick, this cavity has realized a NEP of down to 60 μPa Hz^−1/2^ at 10 kHz and a flat response in the frequency range of 0.2 kHz–22 kHz. Xu et al. further reduced the NEP to 14.5 μPa Hz^−1/2^ using a silver film with higher reflectivity^62^. Figure 4a shows a schematic diagram of the F-P cavity ultrasound sensor with a silver film. Ultrasound sensors made from polymer films that have smaller Young’s modulus have also been used to boost the response to ultrasound. Ultrasound sensors made from 353ND^63^ and polytetrafluoroethylene (PTFE)^64^ films have been used for ranging using the TOF method, with resolutions of 5 mm and 3.7 mm respectively. Figure 4b shows an ultrasound reconstruction of a Plexiglas block in water using a PTFE diaphragm F-P cavity. A microbubble has also been employed for ultrasound sensing as illustrated in Fig. 4c^65^. The microbubble was generated photothermally on a microstructured optical fiber tip, creating a flexible F-P cavity whose gas-water interface was sensitive to ultrasonic waves. This microbubble was capable of detecting weak ultrasounds emitted from red blood cells irradiated by pulsed laser light. Figure 4d shows the reconstructed cross-section photoacoustic image of the blood-filled tubes using this microbubble cavity. This approach can be achieved through sensitivity enhancement of the microbubble as well as the ultrasound response over a certain bandwidth, as shown in Fig. 4e, f. Additionally, owing to its spherical shape and much smaller size than the acoustic wavelength, the 10 μm diameter microbubble has a nearly omnidirectional response, as illustrated in Fig. 4g. To improve the chemical stability of the film and simplify the fabrication process, Fan et al. created an F-P cavity by splicing three sections of cleaved standard single-mode fibers with an off-core cross-section in the middle^66^. This multi-mode dual-cavity F-P interferometer ultrasound sensor has achieved a broadband ultrasound response from 5 kHz to 45.4 MHz.

**Fig. 5:**
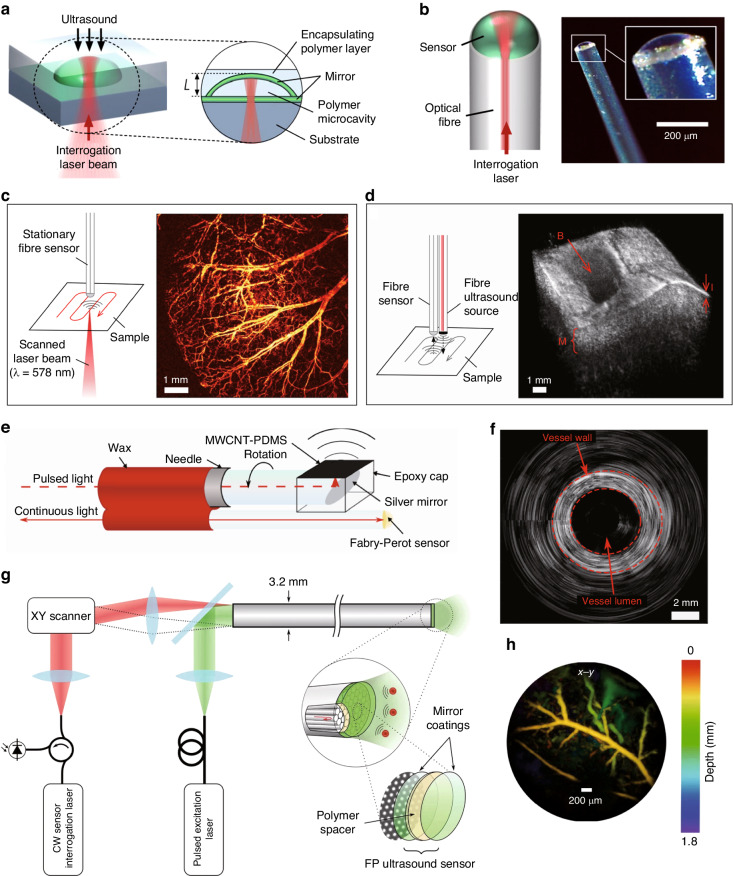
F-P cavity ultrasound sensors for photoacoustic imaging. **a** Plano-concave optical microresonator ultrasound sensor. **b** Optical fiber plano-concave optical microresonator ultrasound sensor. **c** Schematic of fiber-microresonator-sensor based optical-resolution photoacoustic microscopy experiment and image of mouse ear vasculatures in vivo. **d** Schematic of the all-fiber pulse-echo ultrasound experiment and a 3D pulse-echo ultrasound image of ex vivo porcine aorta. **e** Schematic of the side-view optical ultrasound transducer. **f** Rotational optical ultrasound images of an ex vivo swine carotid artery. **g** All-optical forward-viewing photoacoustic endoscopy probe. **h** Photoacoustic image of mouse abdominal skin microvasculature. Reprint (**a**–**d**) from ref. ^52^; Adapted (**e**–**f**) from ref. ^67^; Reprint (**g**–**h**) from ref. ^50^

The above-mentioned F-P cavities utilized air as the cavity medium, which is not ideal for encapsulation and is less robust. To remedy this issue, Guggenheim et al. proposed a plano-concave polymer microresonator formed between two highly reflective mirrors in 2017 (Fig. 5a)^52^. With a high optical *Q* factor of > 10^5^, it exhibited a broadband response of 40 MHz and a NEP of 1.6 mPa Hz^−1/2^. The sensor’s angular response was almost full when integrated on the end face of a fiber (Fig. 5b), rendering it useful as a versatile probe for various applications. Figure 5c, d show an optical-resolution photoacoustic microscopy image of an in vivo mouse ear and a 3D high-resolution pulse-echo ultrasound images of an ex vivo porcine aorta sample, both obtained using this ultrasound sensor on a fiber. Another great advantage of the sensor on the fiber is that it can penetrate deep into the tissue for endoscopic imaging. Additionally, an all-optical rotational B-mode pulse-echo ultrasound imaging system was demonstrated by Colchester et al. using an optical head at the distal end with a multi-walled carbon nanotube and polydimethylsiloxane composite coating (Fig. 5e)^67^. The coating produced axial ultrasound waves via the photoacoustic effect of the light pulses while the F-P cavity next to it can receive the tissue echoes, therefore proving a compact and minimally invasive probing. Figure 5f shows rotational optical ultrasound images of an ex vivo swine carotid artery obtained using this system.

The cladding-core structure of the fibers enables the facile construction of sensing arrays using F-P cavities^68^. In 2018, Ansari et al. has realized a forward-viewing endoscopic probe using a 3.2 mm diameter fiber bundle composed of 50,000 cores, as shown in Fig. 5g^50^. A 15 μm-thick Parylene C film layer sandwiched by two 90% reflective dielectric mirrors was deposited on the end face of the fiber to form the F-P cavity. The large illuminated field of view provided by the excitation laser from all channels allows photoacoustic tomography imaging. Meanwhile, the interrogation laser beam is scanned using a lens and coupled into different fiber cores to read out the ultrasound signals at different locations. The on-axis lateral resolution of the probe was depth-dependent, ranging from 45 to 170 μm for depths between 1 mm and 7 mm, and the vertical resolution was 31 μm over the same depth range. Figure 5h shows the photoacoustic image of a mouse abdominal skin microvasculature. However, the F-P cavities in different channels may have different resonance wavelengths, which poses a challenge for optical readout. To address this issue, Yang et al. demonstrated a photothermal tunable fiber optic ultrasound sensor array, where the resonant wavelength of each cavity can be controlled by a laser^69^. Furthermore, Ma et al. proposed a 4 × 16 fiber-optic array based on F-P cavities, which enabled parallel sensing for imaging with a volume rate of 10 Hz^70^. Moreover, this device’s imaging performance was characterized by reconstructing arbitrary-shaped ultrasound transducer images from the multichannel signals without mechanical scanning.

**Fig. 6:**
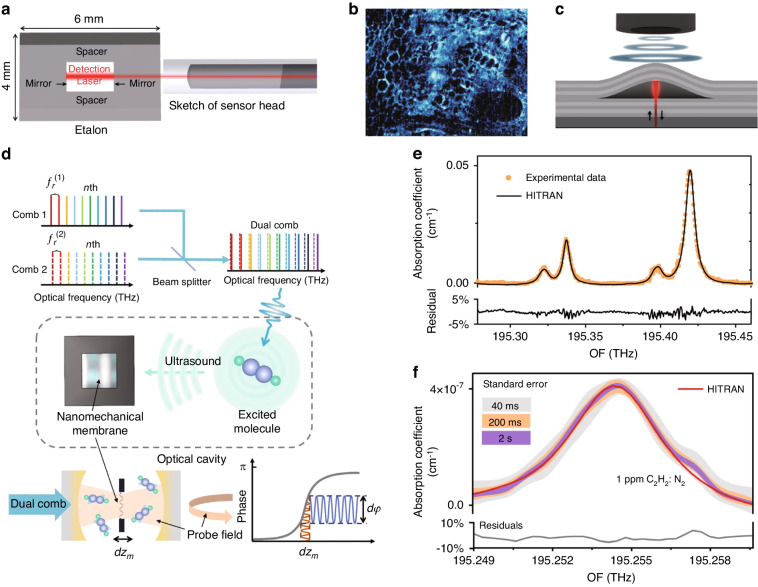
Novel F-P cavities for ultrasound sensing. **a** A miniaturized all-optical akinetic detector based on a rigid F-P resonator. **b** Photoacoustic images of Feulgen-stained Allium Cepa histology samples. **c** Schematic illustration of the buckled-dome ultrasound sensor. **d** The principle of DCOS. **e** Comparison between the DCOS experimental data and the HITRAN model. **f** DCOS enhanced spectra with different acquisition times. Adapted (**a**, **b**) with permission from ref. ^71^ ©Optica Publishing Group; Adapted **c** from ref. ^72^; Reprint (**d**–**f**) from ref. ^73^

In 2016, Preisser et al. demonstrated a novel all-optical akinetic ultrasound sensor using a rigid fiber-coupled F-P etalon with a transparent central opening^71^, as shown in Fig. 6a. Unlike traditional F-P cavity-based ultrasound sensors that rely on measuring the displacement of the cavity mirror, this sensor measures the change in refractive index within the fluid-filled cavity. This unique design resulted in a broadband resonance-free flat response in the 22.5 MHz range, with a sensitivity of 450 μPa Hz^−1/2^. The sensor was successfully employed in photoacoustic imaging of biological samples, as shown in Fig. 6b. Besides being integrated on optical fibers, F-P cavities can also be integrated on a chip. Hornig et al. recently introduced a monolithic buckled-dome cavity for ultrasound sensing, as shown in Fig. 6c. This innovative design achieved an impressive NEP as low as 30–100 μPa Hz^−1/2^ in the frequency range below 5 MHz^72^. Due to the sensitive response of the buckled film to external forces, this device has achieved thermal-noise-limited sensitivity. Moreover, Ren et al. recently developed a technique called dual-comb optomechanical spectroscopy (DCOS) for high-sensitivity ultrasound sensing^73^. Figure 6d illustrates the principle of DCOS, where a dual optical comb is used as the excitation source and an optomechanical coupling system serves as a sensitive photoacoustic detector. Experimental results, displayed in Fig. 6e, f, show a detection limit down to 15 parts per trillion, expanding the range of applications for high-sensitivity ultrasound sensors.

### π-phase-shifted Bragg grating ultrasound sensors

**Fig. 7:**
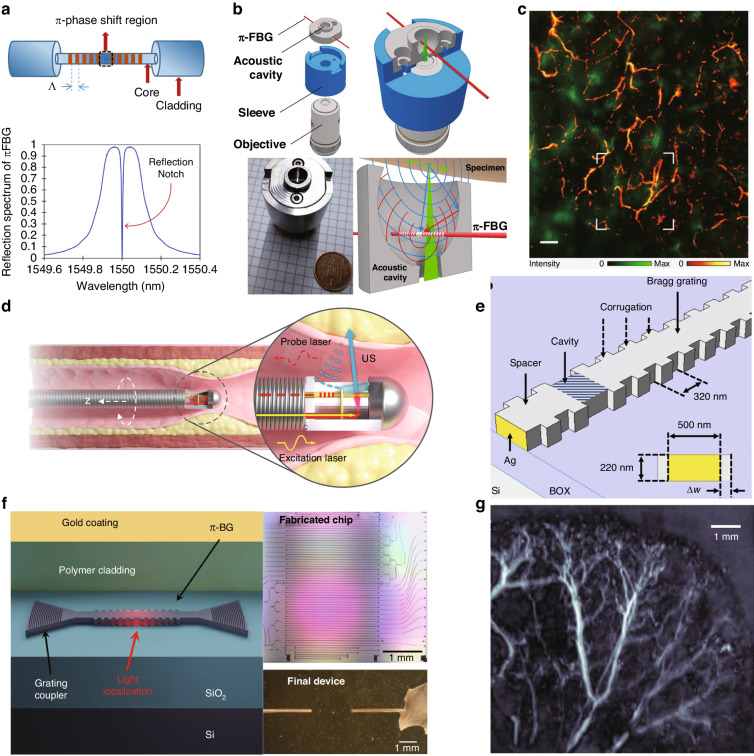
π-phase-shifted Bragg grating ultrasound sensors. **a** Schematic of a π-phase-shifted FBG (upper). The lower figure shows the schematic of the reflection spectrum of a π-FBG. **b** Design and operating principle of the π-FBG-based sensor with an appropriately designed acoustic cavity. **c** Optoacoustic imaging of the lower rear mouse abdomen in vivo, obtained using the π-FBG-based ultrasound sensor in (**b**). **d** Schematic of AO-IVUS imaging of vessel wall. **e** Design of the SWED. **f** Schematic and the optical images of the silicon Bragg grating ultrasound sensors. **g** Maximum intensity projections of the optoacoustic image of a mouse ear. Reprint (**a**) from ref. ^150^; (**b**, **c**) with permission from ref. ^78^ ©Optica Publishing Group; (**d**) from ref. ^79^; ©The Authors, some rights reserved; exclusive licensee AAAS. Distributed under a CC BY-NC 4.0 license http://creativecommons.org/licenses/by-nc/4.0/”. Reprinted with permission from AAAS. **e** from ref. ^80^; (**f**, **g**) from ref. ^81^

The Bragg grating is a structure that has a periodic refractive index. When the Bragg condition is satisfied, there is a high reflectivity in a very small frequency range. Application of an acoustic wave to a Bragg grating alters its effective refractive index and period, thereby modifying the reflectivity of the Bragg grating^74^. However, this approach relies on interference and does not take advantage of optical resonance-enhanced optical readout. Furthermore, accurate detection of ultrasound waves with wavelengths shorter than the length of the grating is limited due to the non-uniformity of their disturbance on the Bragg grating^75^. Consequently, researchers introduced a variation of the π phase at the center of the Bragg grating, creating π-phase-shifted Bragg gratings. This phase jump causes the grating to function as a highly reflective mirror, forming an F-P cavity-like structure within the Bragg grating. Figure 7a illustrates a schematic diagram of a π-BG ultrasound sensor and its reflection spectrum^76^. The formation of the resonator introduces a sharp intensity change in the center of the reflection spectrum (denoted in the reflectivity spectrum in Fig. 7a), significantly amplifying the optical response to ultrasound while reducing the sensing area. In 2011, a π-phase-shifted fiber Bragg grating (π-FBG) with a reflectivity of over 90% was used for ultrasound sensing, achieving a detection frequency range of 10 MHz and a NEP of 440 Pa^22^. Monitoring the shift in the resonance wavelengths was performed using a continuous-wave laser, which was susceptible to laser noise. To improve the sensitivity of the optical readout, Riobó et al. employed a balanced Mach-Zendel interferometer to measure the phase change near the resonance^25^. The adjustment of the interferometric optical path enables the cancellation of the laser’s phase noise, resulting in an SNR that is 24 times higher than conventional intensity measurement methods.

Due to their chip integration capability, π-BGs have great potential for use in bio-imaging. In 2016, Wissmeyer et al. demonstrated the use of a π-FBG in all-optical photoacoustic microscopy, achieving optical resolution in imaging a mouse ear and a zebrafish larva ex vivo^77^. Benefitting from the high optical focusing capability and the wide bandwidth ultrasound inspection capability, the π-FBG has achieved a high lateral resolution of 2.2 μm and an axial resolution of 10.9 μm. π-FBGs can also be effectively combined with optical microscopy to achieve multi-mode imaging. As shown in Fig. 7b, a π-FBG and an acoustic resonant cavity can be compactly integrated, enhancing the ultrasound response while allowing convenient integration with any optical microscope. Shnaiderman et al. utilized this system to achieve in vivo sample measurements in epi-illumination mode, combining optical and optoacoustic microscopy (Fig. 7c)^78^. Similar to F-P cavities on optical fibers, π-FBGs can also be used for endoscopy. Wang et al. reported an all-optical intravascular ultrasound (AO-IVUS) imaging system that utilized picosecond laser pulse-pumped carbon composite for ultrasound excitation and π-FBGs for ultrasound detection (Fig. 7d)^79^. This all-optical technique allowed for ultrawide-bandwidth (147%) and high-resolution (18.6 μm) IVUS imaging, surpassing the capabilities of the conventional techniques.

The integration of π-BGs in chip-integrated waveguides, known as π-phase-shifted waveguide Bragg gratings (π-WBGs), offers additional advantages beyond traditional optical fibers. In a study by Shnaiderman et al., the miniaturization of on-chip integration allowed for a sensing area of 200 nm × 500 nm, with an array of eight sensors^80^. Figure 7e shows the details of this silicon waveguide-etalon detector (SWED). The sensor has achieved a sensitivity of 9 mPa Hz^−1/2^ and a bandwidth of up to 230 MHz. Its remarkable performance enabled imaging of features 50 times smaller than the detected ultrasound wavelength, achieving ultrasound imaging at a resolution comparable to optical microscopy. Another improvement was made to the π-WBG by Hazan et al. in 2022, who coated the grating surface with an elastic medium to eliminate the parasitic effect of surface acoustic waves, as shown in Fig. 7f and ref. ^81^. This silicon-photonics acoustic detector demonstrated an NEP down to 2.2 mPa Hz^−1/2^ and a bandwidth above 200 MHz, corresponding to a theoretically achievable axial resolution of ~ 6 μm. In vivo imaging using this detector was successfully demonstrated for high-resolution optoacoustic tomography, providing imaging of the vasculature of a mouse ear (Fig. 7g).

### Whispering gallery mode microcavity ultrasound sensor

The concept of WGM was first studied in the context of acoustic waves when Lord Reighley made the discovery in the last century that he could hear two people whispering even when they were standing very far away. His study showed that this was due to the continuous reflection along the curved wall with minimal propagation loss. The concept of WGM was later extended to microwaves and optical waves. Analogous to sound waves, light waves can be confined within a closed circular structure of high-refractive-index dielectric material through total internal reflection. When the optical path equals an integer multiple of the optical wavelength, the resonance condition is satisfied^82^. The development of microfabrication technologies in the past few decades has allowed for the realization of WGM microcavities with extremely high optical *Q* factors^83–86^. In addition to their high optical *Q* factors, these microcavities also possess advantages such as small mode volumes, and adaptability to various material systems^87–89^ and shape. As a result WGM optical microcavities have found applications in diverse sensing fields^34,37,39,90–107^. Notably, significant progress has been made in ultrasound sensing using various types of WGM microcavities in the past decade, due to the exquisitely high sensing precision they offer. In the following, we present the recent advances in ultrasound sensing using WGM microcavities with different geometries, specifically including microrings, microspheres, microbubbles, microdisks, and microtoroids. These types of microcavities have their unique advantages in different applications. Microrings, for example, can be easily integrated on the chip and mass-produced, making them ideal for array sensing. Additionally, microring ultrasound sensors exhibit a large response bandwidth, which is critical for photoacoustic imaging. However, the sensitivity of the microring ultrasound sensors is limited due to deformation difficulties, which can be addressed by using more deformable materials and structures. Another challenge lies in obtaining ultrahigh optical *Q* factors of microrings which is important to enhance the sensitivity. On the other hand, the fabrication of ultrahigh *Q* microspheres and microbubbles is quite straightforward. These microcavities also possess almost full-angle spatial response. Microbubbles, in particular, offer unique advantages of detecting gas and liquid samples due to their hollow structure^108–110^. Achieving a lower detection limit requires improvements in both optical and mechanical *Q* factors. Microdisks and microtoroids, with their suspended structure, can significantly enhance mechanical *Q* factors and have become an excellent platform for optomechanics research^111,112^ and have been used for improving ultrasound sensitivity.

#### Microring cavity ultrasound sensors

**Fig. 8:**
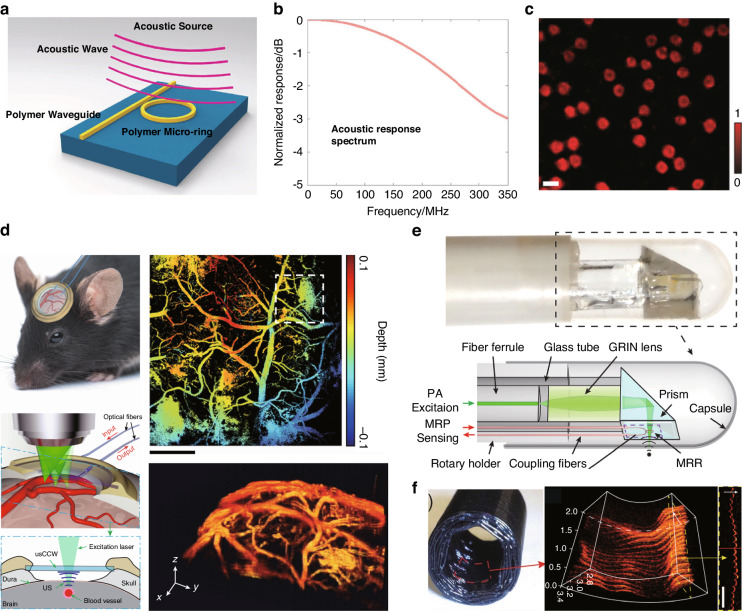
Microring ultrasound sensors. **a** Schematic drawing of the ring working as an acoustic resonator. **b** Frequency domain microring response spectrum. The ring has a −3 dB bandwidth at 350 MHz. **c** Photoacoustic microscopy image of single red blood cells in a mouse blood smear. **d** In vivo photoacoustic microscopy cortical imaging using an ultrasound-sensing chronic cranial window. **e** Photograph and illustration of the microring-based photoacoustic endoscopic probe. **f** Photograph of the black plastic tube phantom(left) and its 3D photoacoustic volumetric rendering of its inner surface(right). Adapted (**a**, **b**) from ref. ^49^; Reprint **c** with permission from ref. ^119^ ©Optica Publishing Group; Reprint (**d**) from ref. ^120^; Reprint (**e**, **f**) with permission from ref. ^121^ © Optica Publishing Group

Microring is one of the most used types of WGM microcavities for ultrasound sensing, due to their integration capability and the availability of various materials options. Typically, microrings are directly sitting on the substrate, making it difficult for an ultrasound to modulate the cavity length. To overcome this limitation, polymer materials with low Young’s modulus, are often chosen to increase strain and improve the response to ultrasound, as shown in Fig. 8a. Some polymer materials such as polymethyl methacrylate (PMMA)^113,114^ and SU-8^115^, can be directly patterned using electron beam lithography (EBL). However, the optical *Q* factors of these microrings are typically limited to the range of 10^3^–10^4^. To improve the optical *Q* factors and thus improve their ultrasound sensitivity, Zhang et al. utilized a nanoimprinting method with silicon molds to fabricate polystyrene (PS) microrings. Through optimization of the nanoimprinting process, they were able to significantly increase the *Q* factor of the polymer microrings to 10^5^ ^116^. Using this high-*Q* PS microring cavity, they have achieved a broadband response of 350 MHz (Fig. 8b) with a NEP of 105 Pa in this frequency range. Such a large response bandwidth allowed them to achieve sub-3 μm axial resolution in photoacoustic imaging^49^. Additionally, they have also explored the potential of an ultrasound sensing array by creating a one-dimensional array consisting of four microrings coupled with a single waveguide^117^.

In 2011, polymer microrings were already being used in photoacoustic imaging, offering a lateral resolution of 5 μm and an axial resolution of 8 μm^118^. Using a polymer microring on a microscope coverslip, Li et al. developed an optically transparent ultrasound detector in 2014^115^. This ultrasound detector offered high-sensitivity over a wide receiving angle, with a bandwidth of 140 MHz and an estimated NEP of 6.8 Pa. The axial resolution was verified to be 5.3 μm through photoacoustic imaging of a carbon-black thin-film target. In 2015, they further improved the system to achieve photoacoustic imaging of mouse erythrocytes, with an axial resolution of 2.1 μm (Fig. 8c)^119^. In 2019, Li et al. reported the development of a disposable ultrasound-sensing chronic cranial window featuring an integrated transparent nanophotonic ultrasound detector^120^. This detector was used to demonstrate photoacoustic microscopy of the cortical vascular network in live mice for 28 days, as shown in Fig. 8d. The small size of the microring also makes it suitable for use as a probe for endoscopy. Dong et al. successfully attached optically transparent polymer microrings and prisms, creating a compact structure where excitation and detection are integrated^121^, as shown in Fig. 8e. By roating the probe, they were able to achieve 3D photoacoustic imaging of the inner wall of a black plastic tube as well as the hair, with the image shown in Fig. 8f.

**Fig. 9:**
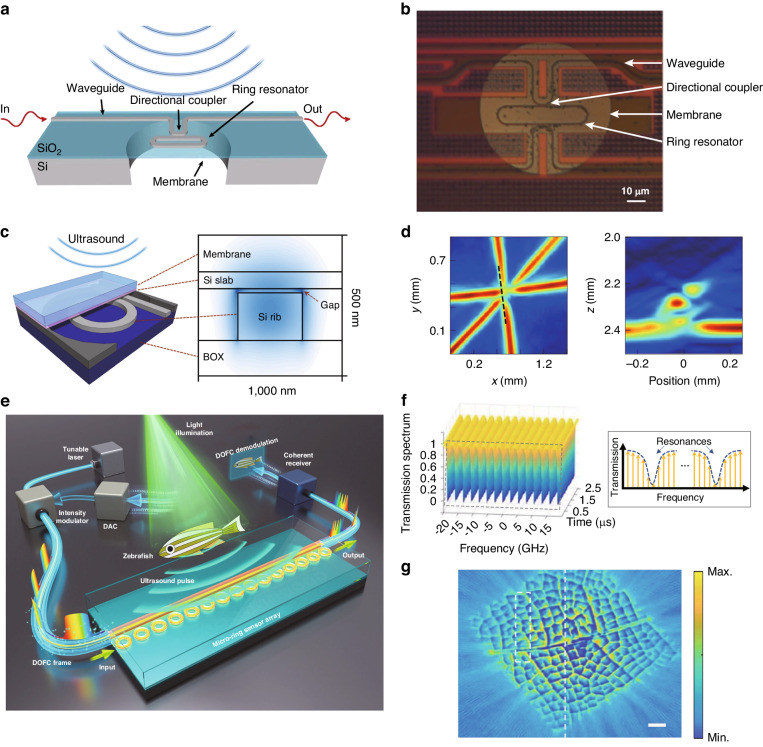
Novel microring cavities for ultrasound sensing. **a** Sketch of the optical micro-machined ultrasound sensor (OMUS), showing the photonic micro-ring resonator on top of the membrane. **b** Microscopic image of an OMUS, showing a membrane with an optical resonator and two directional couplers. **c** Schematic of the optomechanical ultrasound sensor, consisting of a thin film coupled with a silicon microring resonator. **d** 3D photoacoustic tomographic reconstructions of a phantom consisting of three overlaying polyamide structures. **e** Schematic illustration of performing PAT with the chalcogenide-based microring sensor array. **f** Measured transmission spectrum of the sensor array as a function of time in the null case. **g** Imaging result of a piece of leaf buried inside 5-mm thick tissue-mimicking phantoms using the microring sensor array. Reprint (**a**, **b**) from ref. ^122^; Reprint (**c**, **d**) from ref. ^125^; Adapted **e**-**g** from ref. ^128^

Silicon microrings have also been widely utilized for ultrasound sensing, due to the advancements in silicon photonics technology over the past few decades. The technology has enabled cost-effective and mass production of silicon microring cavities on silicon-on-insulator (SOI) platforms. To increase the response of the silicon microring to ultrasound, optical micro-machined ultrasound sensors based on acoustic membranes have been developed. This involves the microfabrication process of etching away the silicon substrate underneath the silicon microring^122,123^, resulting in an increase in mechanical compliance, with the schematic and the optical microscope image of the structure shown in Fig. 9a, b. This suspended silicon membrane has successfully achieved ultrasound pressure as low as 0.4 Pa^122^. However, the deformation of the coupling region can lead to a nonlinear readout. To address this issue, Yang et al. have proposed a solution by partially etching the silicon substrate under the microring region, which maintains a linear readout^124^. In 2021, Westerveld et al. fabricated a thin silicon film over a silicon microring with a 15 nm air gap between them, with the structure illustrated in Fig. 9c. Ultrasound can induce the thin film to vibrate, which changes a change in the air gap and thus affects the intracavity optical field of the microring. Using a microring with a diameter of 20 μm, an NEP of 1.3 mPa Hz^−1/2^ has been realized in the 3 MHz–30 MHz frequency range, which is dominated by acoustomechanical noise^125^. Figure 9d shows the results of the 3D photoacoustic imaging of a phantom consisting of three overlaying polyamide structures, obtained using this ultrasound sensor. They have also designed a one-dimensional array of ten microrings with uniformly distributed resonance wavlengths over a free spectral range of 17 nm. The feasibility of the array detection was verified by measuring the delay in the response of different microrings to the ultrasound. Additionally, there are other microring-like structures such as microknots that can be used for ultrasound sensing^126,127^. In a recent study, Pan et al. demonstrated photoacoustic tomography (PAT) with a chalcogenide-based microring sensor array consisting 15 elements (Fig. 9e). They further developed a parallel interrogation technique using a digital optical frequency comb, as shown in Fig. 9f. They exploited the strong photosensitivity effect of chalcogenide glass and illuminated the microring sensors with pulsed light. By controlling the illumination intensity and duration, they achieved equidistant reconfiguration of the resonance frequencies of the microring sensors. The reconstructed image of the leaf is depicted in Fig. 9g, showing its potential applications of the ultrasound sensor array^128^.

#### Microsphere cavity ultrasound sensors

**Fig. 10:**
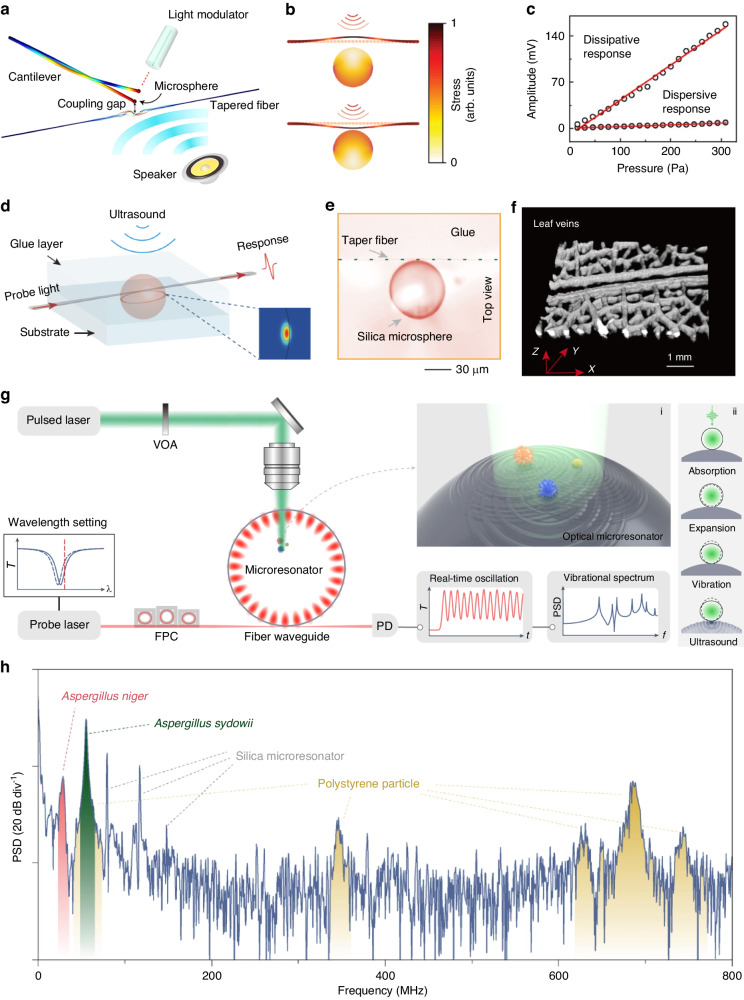
Microsphere ultrasound sensors. **a** Schematic illustration of the mechanical modes of the cantilever-microsphere coupled structure, excited by a temporally-modulated laser beam and a sound wave. **b** Stress field distribution of the microfiber and microcavity as acoustic waves propagate to the coupling system. **c** Dispersive and dissipative acoustic responses at different acoustic pressures. **d** Schematic of microsphere cavity for ultrasound detection. Inset: the cross-section electrical field distribution of a representative WGM mode of the microsphere. **e** The microscopic picture of a silica microsphere cavity. **f** 3D photoacoustic imaging result of leaf veins. **g** Microresonator-based vibrational spectroscopy experimental apparatus. Inset i: the enlarged view of vibrating particles on the optical microresonator. Inset ii: photoacoustic excitation of natural vibrations and their acoustic coupling to the optical mode (from top to bottom). **h** Vibrational spectra of mixed particles. Reprint (**a**) from ref. ^131^; **b**, **c** from ref. ^26^; (**d**–**f**) from ref. ^132^; **g**, **h** from ref. ^151^

The microsphere is another commonly used device geometry due to its easy fabrication process. Silica microspheres, for example, can be fabricated by melting the end of a fiber tip using a CO_2_ laser or a fusion splicer and were extensively utilized for ultrasound sensing^113,129^. In 2014, Chistiakova et al. performed ultrasound sensing in water using an ultra-high *Q* silica microsphere^130^. Through simulations and experimental verification, they demonstrated that the microspheres can detect echo signals from steel balls and water tanks. In 2020, Yang et al demonstrated an optomechanical microdevice based on Brillouin lasing in a microsphere cavity as a sensitive unit for sensing external light, sound, and microwave signals within the same platform^131^, with the structure shown in Fig. 10a. They achieved a NEP of 267 μPa Hz^−1/2^, corresponding to a minimum detectable force of 10 pN Hz^−1/2^. To enhance the sensitivity, they utilized the mechanical vibration modes of the fiber which is coupled to the suspended microsphere. Light is coupled into the microcavity via a thin fiber taper, and the coupling strength relies heavily on the distance between the fiber taper and the microcavity. As ultrasound causes a more significant displacement of the fiber taper compared to the microsphere, measuring the change in the spacing between the fiber taper and the microsphere becomes an effective detection mechanism as shown in Fig. 10b. This dissipative coupling mechanism was further explored using microspheres by Meng et al.^26^, in which they revealed that the response to ultrasound through dissipative coupling was two orders of magnitude higher than the dispersive coupling mechanism (Fig. 10c). In order to create a more compact and environmentally robust microsphere ultrasound sensor, Sun et al. encapsulated the microspheres and fibers using glue, thus preventing contamination^132^, with its schematic illustration and the optical microscope image of the sensor after the encapsulation shown in Fig. 10d,e. The sensor achieved a NEP as low as 160 Pa at 20 MHz, with ultrasound response extending up to 70 MHz. They have successfully applied this sensor in a 3D photoacoustic imaging of leaf veins, with the image shown in Fig. 10f. In 2023, they further extended the application scenarios by integrating microsphere cavities on optical fibers to form microprobes^133^. Additionally, ultrasound sensing in underwater environments has been demonstrated using packaged microspheres^134^. In 2023, Tang et al. demonstrated the use of microsphere ultrasound sensors for real-time vibrational spectroscopy of single mesoscopic particles. As shown in Fig. 10g, the mesoscopic particles deposited on the microsphere generate ultrasound waves when irradiated by a pulsed laser through the photoacoustic effect. The ultrasound waves propagate within the microsphere, which can then excite its mechanical modes. A continuous-wave probe laser is used to couple light into the optical WGM to read out the mechanical motion of the microsphere. The laser wavelength is slightly detuned from the optical resonance so that the mechanical motion can induce a change in the intracavity optical field intensity, which is recorded by a photodetector. They also applied this technology for the biomechanical fingerprinting of microbial cells with different species and living states (Fig. 10h).

#### Microbubble cavity ultrasound sensors

**Fig. 11:**
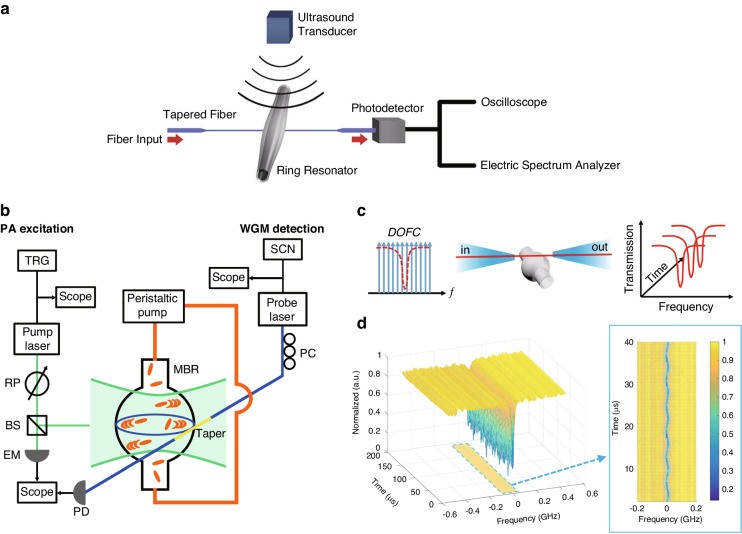
Microbubble ultrasound sensors. **a** Schematic of the experimental setup for ultrasound detection using a microbubble. **b** Schematic of the experimental setup to detect the photoacoustic signal generated by plasmonic nanoparticles. **c** Schematic of the experiments based on digital optical frequency comb methods. **d** Intensity responses in the microbubble-based digital optical frequency comb with ultrasonic stimulation. The insets on the right part are enlarged contour parts. Reprint (**a**) from ref. ^135^; (**b**) from ref. ^138^; (**c**, **d**) from ref. ^140^

Both microrings and microspheres are solid microcavities that are more resistant to deformation compared to hollow structures. Consequently, microbubble cavities fabricated using hollow capillaries have been widely utilized for ultrasound sensing. The capillary walls can be crafted to be exceptionally thin to amplify the ultrasound response. In 2017, Kim et al. developed a microbubble-based ultrasound sensor (Fig. 11a) that has reached a NEP of 215 mPa Hz^−1/2^ and 41 mPa Hz^−1/2^ at 50 kHz and 800 kHz in air, respectively^135^. Microbubbles also employ fiber tapers for light coupling and need to be encapsulated in complex detection environments. Tu et al. used an encapsulated microbubble to detect acoustic waves at low frequencies in the 10 Hz to 100 kHz range, achieving a NEP of 2.2 mPa Hz^−1/2^ ^136^. Benefiting from their encapsulated structure, microbubble sensors maintain stable performance under varying temperatures and static pressures. A unique advantage of microbubbles over other microcavities is that their walls can serve as ultrasound transducers, while the hollow structure inside can act as a sample container. In recent years, various studies^137–139^ have explored the use of nanoparticles injected into microbubbles for photoacoustic detection of flowing samples, as depicted in Fig. 11b. This approach allows non-contact detection of target particles and can distinguish the optical absorption spectra between different particles. Most recently in 2020, Pan et al. used a microbubble cavity combined with a digital optical frequency comb for ultrasound detection in air, which allows for capturing the full mode spectrum on a microsecond timescale. The working principle and experimental results of this work are shown in Fig. 11c,d^140^. They have achieved a NEP of 4.4 mPa Hz^−1/2^ in the air at a frequency of 165 kHz and also accomplished high positioning precision by measuring the phase difference between two microbubbles. Optical frequency combs were also used in microrings on the chip for ultrasound measurement^141^.

#### Microdisk and microtoroid cavity ultrasound sensors

**Fig. 12:**
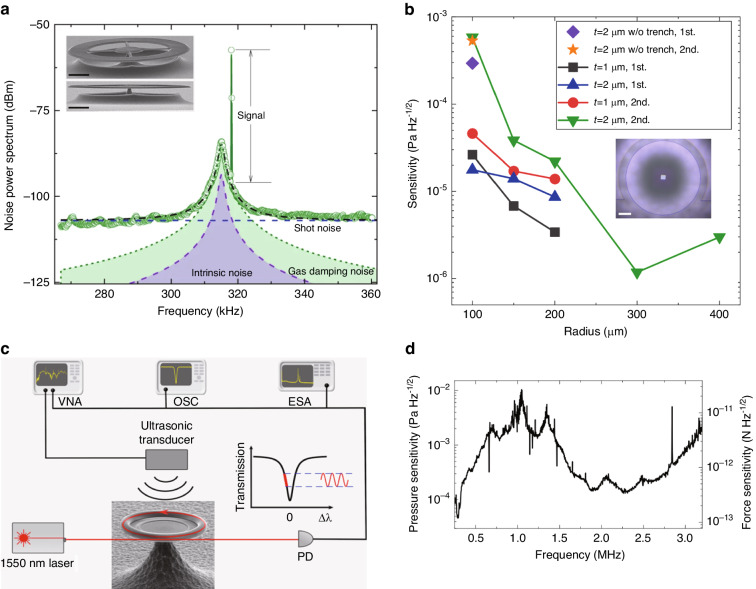
Microdisks and microtoroids ultrasound sensors. **a** Noise power spectrum of the sensor near a mechanical mode of the microdisk. Inset: SEM picture of a suspended spoked microdisk. **b** Sensitivities at the flapping modes of microdisks with different thicknesses and radii. Inset: Top-view optical microscope image of a microdisk with a trench structure. **c** Schematic diagram of the experimental setup for ultrasound sensing using a microtoroid. **d** Sensitivity spectra of the microtoroid ultrasound sensor. Adapted (**a**) from ref. ^41^; Adapted **b** from ref. ^42^. Reprint (**c**, **d**) from ref. ^143^

In addition to the three common types of WGM microcavities mentioned above, microdisks have also been utilized for ultrasound sensing. The microdisk structure offers several advantages. First, advanced microfabrication techniques allow for the creation of large sensing areas, thus improving the sensitivity. Second, the design of suspended microdisk structures augments mechanical compliance to enhance ultrasound response, and decrease mechanical damping rate *γ*, allowing for improved thermal-noise-limited sensitivity (Eq. (3)). In 2019, Basiri-Esfahani et al. demonstrated an ultrasound sensor using a suspended spoked microdisk and reached the noise region dominated by collisions of gas molecules^41^. The spoke structure can make the microdisk more mechanically compliant, reducing mechanical losses and making it easier to reach the thermal-noise-limited regime. Figure 12a shows the noise power spectrum (black) of the microdisk around the mechanical mode, as well as its ultrasound response at a single frequency (green curve). This allowed NEPs of 8–300 μPa Hz^−1/2^ at a frequency range between 1 kHz and 1 MHz. They used both dissipative and dispersive mechanisms to read out the different mechanical vibration modes. This study demonstrated a significant improvement in the sensitivity of ultrasound sensors in the range dominated by thermal noise. In 2023, Yang et al. performed a more systematic study on the thermal-noise-limited ultrasound sensitivity using suspended microdisks, both theoretically and experimentally^42^. The sensitivity was optimized by varying the radius and thickness of the microdisk, as well as using a trench structure around the disk. Sensitivities of microdisks with different thicknesses and radii are shown in Fig. 12b. A peak sensitivity of 1.18 μPa Hz^−1/2^ has been realized at 82.6 kHz, using a microdisk with a radius of 300 μm and a thickness of 2 μm. In the same year, Xing et al. utilized an ultrahigh-quality calcium fluoride resonator for ultrasound sensing, reaching a sensitivity of 9.4 μPa Hz^−1/2^ at 10 kHz^142^.

The optical *Q* factors of microdisks can be significantly enhanced by converting the microdisk into a microtoroid. This transformation involves a process of melting the microdisk edges, thereby creating a microtoroid with an incredibly smooth surface. Exceptionally high *Q* factors of up to 10^8^ have been achieved using this method^83^. To further expand the frequency range of air-coupled ultrasound detection, Yang et al. then used microtoroids to improve the megahertz-frequency ultrasound detection^143^, with their ultrasound measurement setup shown in Fig. 12c. By employing a microtoroid with a very thin silicon pedestal, an impressive mechanical *Q* factor of 700 was attained for the first-order flapping mode at 2.56 MHz. Figure 12d displays the pressure and force sensitivities on the left and right axes, respectively. Near the mechanical resonance, thermal-noise-limited sensitivity has been achieved in a frequency range of 0.6 MHz. Sensitivities of 46 μPa Hz^−1/2^-10 mPa Hz^−1/2^ have been realized in the frequency range of 0.25–3.2 MHz.

### Performance comparison

Table 1 presents a summary of the key parameters for three types of optical microcavity-based ultrasound sensors, including the optical *Q* factor, center frequency, bandwidth, NEPD or NEP, and acceptance angle. F-P cavities can achieve an optical *Q* factor in the range of about 10^4^ to 10^5^, by utilizing a highly reflective dielectric layer. Similarly, π-BGs exhibit optical *Q* factors at a comparable level, which can be further increased by increasing the grating length. WGM microcavities, especially microspheres, microbubbles, and microtoroids, can achieve higher optical *Q* factors, typically ranging from 10^7^ to 10^8^. For microrings, selecting materials with lower optical absorption losses, such as silicon nitride, can improve the optical *Q* factors^144^. Higher optical *Q* factors enable the attainment of the thermal-noise-limited sensitivity, leading to a lower NEP. Most of the sensors with NEP at the micropascal level have exploited the mechanical resonances of the structures to achieve the thermal-noise-limited sensitivity, except for a few millimeter-scale F-P cavities. However, it is important to note that mechanical resonances can limit the detection bandwidth, which can pose challenges in certain applications. For most imaging applications, bandwidth at the megahertz level is necessary. Nevertheless, due to the large propagation loss of high-frequency ultrasound waves, only a limited number of sensors can detect megahertz frequency ultrasound in water. Air-coupled high-sensitivity ultrasound detection above 1 MHz frequency was only realized using a microtoroid cavity, primarily due to the higher absorption loss in air. Sensors integrated into optical fibers generally have wider acceptance angles, making them suitable for receiving ultrasound signals from various directions. Additionally, optical fibers themselves serve as excellent transmission devices and can be easily connected to external devices such as lasers. Sensors integrated onto a chip have slightly narrower acceptance angles but offer advantages of low cost, low power consumption, and mass production. However, stand-alone sensors face the challenge of ensuring stable packaging for practical applications beyond laboratory settings. For 2D and 3D imaging, the use of multiple sensor arrays working simultaneously can reduce the need for mechanical moving parts and expedite the imaging process. While 2D array multi-channel parallel sensing has already been achieved with F-P cavities on optical fibers, the development of array sensing is still in its infancy, as only one-dimensional arrays of π-BGs and WGM microcavities have been demonstrated thus far.

**Table 1:** Summary of the performances of optical ultrasound detection with different microcavities

	Structure	*Q* factor	CF (MHz)	BW at −6 dB (MHz)	NEPD (mPa Hz^−1/2^)	Acceptance angle
FP	Diaphragm^61^	-	0.01	0.022	0.060	Around 80^∘^
	F-P etalon^71^	-	1	22.5	0.450	< 20^∘^
	Plano-concave microresonator^52^	>10^5^	3.5	>20	2.1	180^∘^
	Microbubble F-P cavity^65^	-	0.7	0.8	3.4	180^∘^
	Buckled-dome microcavities^72^	~ 10^3^	-	>15	0.03–0.1	120^∘^
π-BG	FBG^22^	2 × 10^5^	6.5	3	(NEP) 440 Pa	-
	FBG^78^	1.9 × 10^5^	~ 25	~ 36	(NEP) 88 Pa	153^∘^
	FBG^79^	-	27.5	40.4	(NEP) 108 Pa	-
	WBG^80^	~ 10^5^	-	230	9	148^∘^
	WBG^81^	-	-	200	2.2	-
WGM	Microring^49^	~ 10^5^	-	350 (−3 dB)	(NEP) 105 Pa	-
	Microring^119^	~ 10^4^	-	280	(NEP) 6.8 Pa	14^∘^
	Microring^122^	~ 10^4^	0.76	0.14	(NEP) 0.4 Pa	-
	Microring^125^	~ 10^4^	-	27	1.3	120^∘^
	Microring^128^	~ 10^5^	-	175	2.2	60^∘^
	Microsphere^130^	9.5 × 10^7^	40	5	(NEP) 0.535 Pa	-
	Microsphere^131^	10^8^	0.0057	-	0.267	-
	Microsphere^132^	~ 10^5^	20	70	(NEP) 100 Pa	-
	Microsphere^26^	~ 10^6^	0.14	-	1.29	-
	Microsphere^133^	~ 10^6^	~ 30	150	1.07	180^∘^
	Microbubble^135^	3.5 × 10^7^	0.8	0.2	41	-
	Microbubble^140^	3 × 10^7^	0.165	-	4.4	-
	Microbubble^136^	5.2 × 10^5^	0.001	0.1	2.2	105.5^∘^
	Microdisk^41^	3.6 × 10^6^	0.318	-	0.008–0.3	-
	Microdisk^42^	3 × 10^6^	0.0826	-	0.00118	-
	Microdisk^142^	1.02 × 10^8^	0.01	0.02	0.0094	150^∘^
	Microtoroid^143^	~ 10^7^	2.56	1.13	0.046-10	-

*CF* center frequency, *BW* bandwidth

## Conclusion

Over the past few decades, optical ultrasound sensors have emerged as a promising alternative to traditional piezoelectric sensors, offering superior sensitivity and bandwidth. Among these, optical microcavity ultrasound sensors have particularly stood out due to their high sensitivity, broad bandwidth, and miniaturization capabilities, making them suitable for a wide range of applications in ultrasound imaging and photoacoustic sensing. This review aims to explore the advancements in ultrasound sensing utilizing optical microcavities. We first introduce the sensing principles and readout mechanisms, highlighting the key parameters of microcavity ultrasound sensors. Previous work has shown that thermal noise is the fundamental limitation of NEP, and in this review, we have discussed the parameters that influence the sensor response and sensitivity. Furthermore, we provide a comprehensive overview of the works on ultrasound sensing using three different types of optical microcavities, including F-P cavities, π-phase-shifted Bragg gratings, and WGM microcavities.

Additionally, we compare the performance of these microcavity ultrasound sensors. F-P cavity-based ultrasound sensors demonstrate low NEPs but necessitate suspended thin film structures and possess relatively large sensing areas. In contrast, solid F-P cavities offer inferior sensitivities but broader response bandwidth. Fiber-based F-P cavities allow almost full spatial angle response and multi-channel parallel sensing. π-BGs exhibit advantages such as broadband response, large acceptance angle, multi-parameter sensing, and ease of on-chip integration, yet sensitivity improvement is necessary. WGM microcavities can achieve higher sensitivities, due to the higher optical and mechanical *Q* factors which allow thermal-noise-limited sensitivities to be reached. However, WGM microcavities are not yet commercially mature for several reasons. Firstly, for suspended WGM microcavities (e.g., microspheres and microdisks), the often-used fiber taper couplers are challenging to integrate. Secondly, on-chip integrated WGM microcavities are less demanding in terms of packaging, but their fabrication process is highly intricate. Achieving high *Q* optical microcavities requires the use of high-precision fabrication techniques, such as electron-beam lithography and deep-ultraviolet photolithography. However, these methods, despite their effectiveness, are impractical for industrial applications due to their prohibitive costs. Besides, utilizing these microcavities necessitates extra intricate and costly equipment for the measurement setup, limiting its application scenarios. Expectantly, some researchers have made attempts at portable measurement systems, such as a phone-sized microresonator sensing system that can be equipped on a drone^145,146^. Looking forward, advancements in both science and technology are anticipated to enhance the performance of optical microcavity ultrasound sensors, resulting in lower NEPs, broader bandwidths, and larger acceptance angles. Moreover, their potential for parallel sensing requires further exploration to enable high-speed imaging^147^ and sensing applications. One approach is the combination of multi-wavelength frequency comb sources^148,149^ with an ultrasound sensor array^128^. By harnessing these advancements, optical microcavities hold promise to revolutionize ultrasound sensing in numerous applications, including photoacoustic imaging, non-destructive detection, mineral exploration, underwater communications, etc.
